# Mitochondrial Reactive Oxygen Species Regulate Adipocyte Differentiation of Mesenchymal Stem Cells in Hematopoietic Stress Induced by Arabinosylcytosine

**DOI:** 10.1371/journal.pone.0120629

**Published:** 2015-03-13

**Authors:** Weimin Wang, Yao Zhang, Wenyi Lu, Kaiyan Liu

**Affiliations:** 1 Department of Hematology, Peking University People's Hospital, Beijing, China; 2 Institute of Hematology, Peking University, Beijing, China; University of Newcastle, UNITED KINGDOM

## Abstract

**Objective:**

The increase in adipocytes induced by chemotherapeutic drugs may play a negative role in hematopoietic recovery. However, the mechanism underlying adipocyte differentiation of mesenchymal stem cells (MSCs) in hematopoietic stress is still unknown. Hence, the involvement of reactive oxygen species (ROS) in adipocyte differentiation under hematopoietic stress was investigated *in vitro* and *in vivo*.

**Methods:**

The roles of cellular ROS in adipogenesis were investigated *in vivo* through an adipocyte hyperplasia marrow model under hematopoietic stress induced by arabinosylcytosine (Ara-C) and *in vitro* via adipocyte differentiation of human MSCs. ROS levels were detected using the CM-H_2_DCFDA probe and Mito-SOX dye. Adipogenesis was evaluated by histopathology and oil red O staining, whereas detection of mRNA levels of antioxidant enzymes and adipogenesis markers was performed using quantitative real-time polymerase chain reaction analysis.

**Results:**

ROS were found to play an important role in regulating adipocyte differentiation of MSCs by activating peroxisome proliferator-activated receptor gamma (PPARγ,) while the antioxidant N-acetyl-L-cysteine acts through ROS to inhibit adipocyte differentiation. The elevated ROS levels induced by Ara-C were caused by both over-generation of mitochondrial ROS and reduction of antioxidant enzymes (Cu/Zn Superoxide dismutase and catalase). Our findings suggest that a mitochondrial-targeted antioxidant could diminish adipocyte differentiation.

## Introduction

Long-term chemotherapy and hematopoietic stem cell transplantation (HSCT) are effective treatments for hematologic malignancies. Nevertheless, delayed hematopoietic recovery and related complications, including infection, anemia, and hemorrhage, occur in a considerable number of patients and seriously affect patient survival [1]. The reasons and mechanisms of delayed hematopoietic recovery are still not clear. More recently, in addition to hematopoietic stem cells (HSCs), the bone marrow (BM) hematopoietic microenvironment, which represents an important niche for HSCs, has been reported to be impaired after long-term chemotherapy and HSCT [2–6]. Several studies have uncovered the role of adipocytes in actively suppressing hematopoiesis rather than passively filling the space of damaged BM [7,8]. In addition, previous studies by our research group have demonstrated that adipocyte hyperplasia can be induced by arabinosylcytosine (Ara-C) treatment, while bisphenol A diglycidyl ether (BADGE), a peroxisome proliferator-activated receptor gamma inhibitor), contributes to improved hematopoietic recovery after chemotherapy by inhibiting adipogenesis [9]. Therefore, the increase in adipocytes induced by chemotherapeutic drugs may play a negative role in hematopoietic recovery following chemotherapy and HSCT. Adipocytes in BM are derived from the differentiation of mesenchymal stem cells (MSCs) [10]. Thus, further understanding of the mechanism underlying adipocyte differentiation from MSCs might facilitate the cognition of adipogenesis induced by chemotherapy.

ROS is a heterogeneous group of molecules that are free radicals derived from diatomic oxygen and exhibit a wide spectrum of reactivity. ROS generated by the mitochondria or nicotinamide adenine dinucleotide phosphate (NADPH) oxidases have been shown to influence cell-cycle progression, cell motility, and growth factor signaling in a variety of normal cell types [11]. Recently, some studies have illustrated that the generation of ROS is not simply a consequence of differentiation, but also a causal factor in promoting adipocyte differentiation [12–14], whereas adipocyte differentiation is diminished in the presence of antioxidants. Moreover, clinical research suggests that chemotherapy with DNA-damaging agents may cause mitochondrial DNA (mtDNA) mutations in primary leukemia cells, which are associated with increased ROS generation [15]. Hence, we speculate that adipogenesis induced by chemotherapy is most likely associated with the ROS level in MSCs. The use of an antioxidant may be a potential way to inhibit adipogenesis that in turn improves hematopoietic recovery.

To confirm our hypothesis, the role of cellular ROS in adipogenesis was investigated *in vivo* through an adipocyte hyperplasia marrow model under hematopoietic stress induced by Ara-C and *in vitro* via adipocyte differentiation of human MSCs. Meanwhile, the effect of the antioxidant NAC on ROS and adipocyte hyperplasia were also studied *in vivo* and *in vitro*. Furthermore, the potential mechanism of the elevated ROS levels induced by Ara-C was investigated.

## Materials and Methods

### Animals

C57BL/6J female mice (6–8 weeks old) were purchased from the Experimental Animal Center of Military Medical Sciences Academy (Beijing, China). Mice were housed in a controlled environment (12 h light/dark cycles at 21°C). Animal experiments were approved by the Animal Ethics Committee of Peking University Health Science Center (permit number: 2013–16). At each time point, mice were euthanized by cervical dislocation under sodium pentobarbital anesthesia (50 mg/kg).

### Animal treatments

Control group animals were injected with the same volume of phosphate-buffered saline (PBS, vehicule) for four consecutive days. Each group (Control, Ara-C, NAC or Ara-C+NAC) was composed of 10 animals. Ara-C group animals were treated by 0.5 g/Kg per day of Ara-C (Sigma, USA) via intraperitoneal injection for four consecutive days to induce hematopoietic stress. The NAC treatment method was performed according with the method published recently by Hu et al [16]. NAC (Sigma, USA) was administered at a dose of 0.1 g/Kg per day via intraperitoneal injection for four consecutive days. The NAC-injected mice also received NAC in their drinking water at a dose of 6 μM until the termination of the experiments to reduce intracellular ROS. Animals in the Ara-C+NAC group were administered both Ara-C and NAC reagents as described above.

### Bone marrow extraction

After the mice were sacrificed, their femurs and tibias were carefully cleaned to remove the adherent soft tissue. Bone marrow was harvested by inserting a syringe needle into one end of the bone and flushing with PBS containing 2% (v/v) fetal bovine serum (FBS; Gibco, USA). Bones were crushed with a scalpel in PBS containing 2% (v/v) FBS. Suspensions were digested with 3 mg/ml collagenase I (Sigma, USA) for 30 minutes, filtered through a 70 μm nylon filter cell strainer (BD, USA) and washed with PBS.

### Cell culture

BM-derived MSCs were isolated by a previously published method with slight modifications [17]. Cells harvested from human BM were seeded in T25 flasks (Corning, USA) with low glucose Dulbecco’s modified Eagle’s medium (DMEM; Gibco, USA) containing 10% (v/v) FBS, 2 mM L-glutamine, 100 U/ml penicillin and 100 μg/ml streptomycin. and were cultured at 37°C with 5% CO_2_. After adhering for three days, the medium was thoroughly replaced to maintain the adherent cells and remove the non-adherent cells. The medium was changed every three days.

### Adipocyte differentiation, NAC treatment and oil red O staining

At passage three, human BM-derived MSCs were cultured with the Adipogenesis Differentiation Kit (Gibco, USA) according to the manufacturer’s instructions. At day 14 of differentiation, oil red O staining was carried out. Briefly, cells were washed with PBS and fixed in 4% paraformaldehyde for 30 minutes. After washing twice with PBS, cells were stained with 0.3% oil red O solution (Sigma Aldrich, USA) for 20 minutes at room temperature. Staining was quantified by extracting oil red O from the stained cells with isopropanol, followed by determining the optical density (OD) values of the solution at 518 nm. The NAC treatment method was performed according to the method published by Tormos et al [13]. The NAC treatment was started on Day 2 of differentiation and lasted until Day 14. On Day 1, the differentiation and NAC+differentiation groups were treated with the Adipogenesis Differentiation Kit, while the undifferentiated and NAC-treated groups were not subjected to adipocyte differentiation. On Day 2, cells were exposed for 4 hours prior to ROS measurement to NAC treatment (5 mM) or to PBS for control group.

### Histopathology

After the mice were sacrificed, tibias were collected followed by fixation in 4% (w/v) paraformaldehyde for 24 h. Tissues were decalcified in 20% (w/v) ethylenediaminetetraacetic acid (EDTA) (pH 7.5) for 7 days at 4°C and then paraffin embedded. Sections (4 μm thick) were mounted on slides, deparaffinized, and stained with hematoxylin and eosin (HE).

### Measurement of cellular ROS

Total and mitochondrial ROS levels were quantified by fluorescence-activated cell sorting (FACS) using the 5-(and-6)-chloromethyl-2,7-dichlorodihydrofluorescein diacetate CM-H2DCFDA probe and MitoSOX dye, respectively (Molecular Probes, USA). Briefly, after trypsinization with 0.25% (w/v) Trypsin–EDTA (Gibco, USA), cells were collected and rinsed with PBS. Cells were then resuspended and incubated in pre-warmed PBS containing 10 μM CM-H2DCFDA or 5 μM MitoSOX in the dark for 20 min at 37°C. Intracellular fluorescence was then quantified using a BD Calibur flow cytometer (Becton Dickinson, USA). For evaluating the contribution of NADH oxidase (NOX) and mitochondria on ROS production induced by Ara-C in vitro, cells were pretreated for 24 h with 100 mM Ara-C and for 1 hour with 500 nM of diphenyleneiodoniumchloride (DPI; Sigma Aldrich, USA), a NOX inhibitor, or 500 nM of the mitochondria-targeting antioxidant Mito-Tempo (Sigma Aldrich, USA). Afterwards, total ROS produced by cells were measured as described above for the CM-H2DCFDA probe.

### Detection of mitochondrial ROS in live cells

ROS produced by mitochondria were detected using a MitoSOX Red superoxide indicator (Molecular Probes, USA) according to the manufacturer’s instructions. Live human MSCs were labeled with MitoSOX Red reagent, which fluoresces when oxidized by superoxide, and nuclei were stained with the blue fluorescent dye Hoechst 33342 (Sigma Aldrich, USA).

### Antioxidant enzyme activity assays and glutathione levels

The activity of superoxide dismutase (SOD) and catalase (CAT) was assessed using the Cu/Zn-SOD and Mn-SOD Assay Kit (Beyotime, China), and the CAT Assay Kit (Beyotime, China) respectively according to the manufacturers’ instructions. Glutathione (GSH) levels were quantified using a Glutathione Assay Kit (Beyotime, China) according to the manufacturer’s instructions.

### Quantitative polymerase chain reaction analysis

Total RNA was isolated from pooled marrow cells or cultured MSCs using TRIzol Reagent (Invitrogen, USA). RNA (1 ug) was reverse-transcribed using a High Capacity cDNA Reverse Transcription Kit (Applied Biosystems, USA) according to the manufacturer’s instructions. Quantitative polymerase chain reaction (qPCR) assays were performed using an ABI 7500 Fast Real-Time PCR System, Power SYBR Green PCR Master Mix (Applied Biosystems, USA), and primers (see Table 1 for primer sequences). Human GAPDH and mouse GAPDH served as endogenous controls. Data were analyzed using 7500 Fast System SDS version 2.0.6 software (Applied Biosystems, USA). Relative quantification of the target genes were normalized to endogenous control levels and calculated with the 2^-ΔΔCt^ method.

**Table 1:** Sequences of primers used for qPCR.

Primers	Sequences (5’→3’)
Human PPARγ, forward	TTCAAGACAACCTGCTACAAG
Human PPARγ, reverse	CGTGTTCCGTGACAATCT
Human adiponectin, forward	GGCTATGCTCTTCACCTATG
Human adiponectin, reverse	TCCATTACGCTCTCCTTCC
Human NOX2, forward	TAACGCCACCAATCTGAAGC
Human NOX2, reverse	CATCCCAGCCAGTGAGGTAG
Human NOX4, forward	GAAGAGCCCAGATTCCAAGC
Human NOX4, reverse	TGACCGAAATGATGGTGACTG
Human MnSOD, forward	CCTGGAACCTCACATCAACG
Human MnSOD, reverse	CCAACGCCTCCTGGTACTTC
Human Cu-ZnSOD, forward	GGGCAATGTGACTGCTGAC
Human Cu-ZnSOD, reverse	ACAAGCCAAACGACTTCCAG
Human CAT, forward	GCCTTTGGCTACTTTGAGGTC
Human CAT, reverse	GAGAACCGAACTGCGATGG
Human GAPDH, forward	AGAAGGCTGGGGCTCATTTG
Human GAPDH, reverse	AGGGGCCATCCACAGTCTTC
Mouse PPARγ, forward	CGAGGACATCCAAGACAAC
Mouse PPARγ, reverse	GTGCTCTGTGACGATCTG
Mouse adiponectin, forward	GCCGCTTATGTGTATCGCTCAG
Mouse adiponectin, reverse	GCCAGTGCTGCCGTCATAATG
Mouse GAPDH, forward	TCAATGACAACTTTGTCAAGCTCA
Mouse GAPDH, reverse	GTGGGTGGTCCAGGGTTTCTTACT

### Immunoblot analysis

Whole cell lysates were obtained using RIPA lysis buffer (Solarbio, China). The protein concentration was determined using the BCA Protein Assay Reagent Kit (Solarbio, China). Equal amounts of samples were separated by sodium dodecyl sulfate-polyacrylamide gel electrophoresis and transferred to polyvinylidene fluoride membrane (Millipore, USA). The membrane was incubated with 5% non-fat skim milk in Tris-Buffered Saline and Tween 20 (TBST) for 2 h, followed by hybridization at 4°C overnight with primary antibodies for PPARγ (rabbit polyclonal, cat no. ab19481, diluted 1:100; Abcam, UK) and GAPDH (rabbit polyclonal, cat no. ab37168, diluted 1:5000; Abcam, UK). After washing with TBST, the membrane was incubated with horseradish peroxidase-conjugated goat anti-rabbit IgG secondary antibody (cat no. sc2004, diluted 1:10000; Santa Cruz, USA) for 1 h. The bands were detected by enhanced chemiluminescence substrate (Thermo Fisher Scientific, USA). Relative expression of the target proteins was normalized to GAPDH levels.

### Statistical analysis

Data are presented as mean ± standard deviation (SD) or mean ± standard error of the mean (SEM). Statistical differences between two groups were evaluated by the Student’s t test. For multiple group comparisons, data were analyzed by one-way analysis of variance (ANOVA). A value of *P* less than 0.05 was considered statistically significant.

## Results

### ROS mediate adipocyte differentiation of MSCs *in vitro*

**Fig 1:**
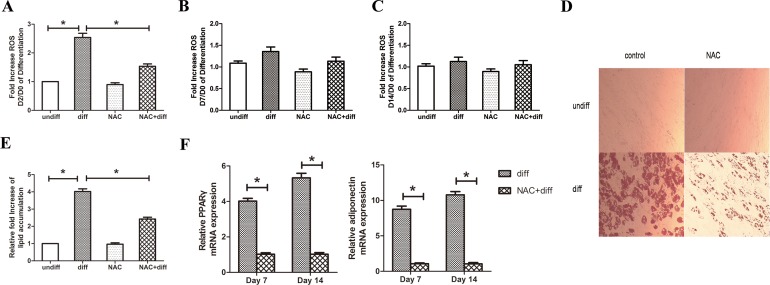
ROS mediate adipocyte differentiation of MSCs *in vitro*. (A) Fold increase in ROS on Day 2/Day 0 of differentiation. “NAC” and “NAC+diff” groups were treated with 5 mM NAC for 4 h prior to ROS measurement on Day 2. The NAC treatment was administered daily and lasted to Day 14. (B) Fold increase in ROS on Day 7/Day 0 of differentiation. (C) Fold increase in ROS on Day 14/Day 0 of differentiation. (D, E) NAC diminished lipid accumulation. Cells were fixed and stained with oil red O on Day 14. The oil red O was extracted with isopropanol and absorbance was measured at 518 nm. (F) Gene expression of PPARγ and adiponectin was decreased in the presence of NAC at Day 7 and Day 14 of differentiation compared to control. *P < 0.05.

To investigate the role of ROS in adipocyte differentiation of MSCs, the change in ROS production during differentiation and the effect of the antioxidant NAC on differentiation *in vitro* was examined. The NAC treatment started on Day 2 of differentiation and lasted until Day 14. Antioxidant treatment began on Day 2 to avoid interference with mitotic clonal expansion during adipocyte differentiation, as previously described [18]. As shown in Fig. 1A, an increase in cellular ROS was observed in human MSCs on Day 2 of differentiation compared to Day 0. And this increase was abolished by treatment with the antioxidant NAC. The change in ROS levels was examined at Day 7 and Day 14. It was found that the generation of ROS in the early stage of differentiation was gradually attenuated at Day 7 and Day 14 (Fig. 1B-C) compared to Day 2, whereas NAC treatment had no effect. After cells were cultured with the Adipogenesis Differentiation Kit for 14 days, a significant increase in fat droplets and lipid accumulation was observed in differentiated cells compared to undifferentiated cells. Meanwhile, an inhibition effect on lipid accumulation was observed after NAC treatment (Fig. 1D-E). In conjunction with lipid accumulation, mRNA levels of the major adipogenic transcription factor PPARγ and its target gene adiponectin were also significantly decreased at Day 7 and Day 14 in the presence of NAC (Fig. 1F). These results indicate that ROS play an important role in regulating adipocyte differentiation of MSCs that can be regulated by the antioxidant NAC.

### NAC diminishes adipogenesis induced by Ara-C *in vivo*

**Fig 2:**
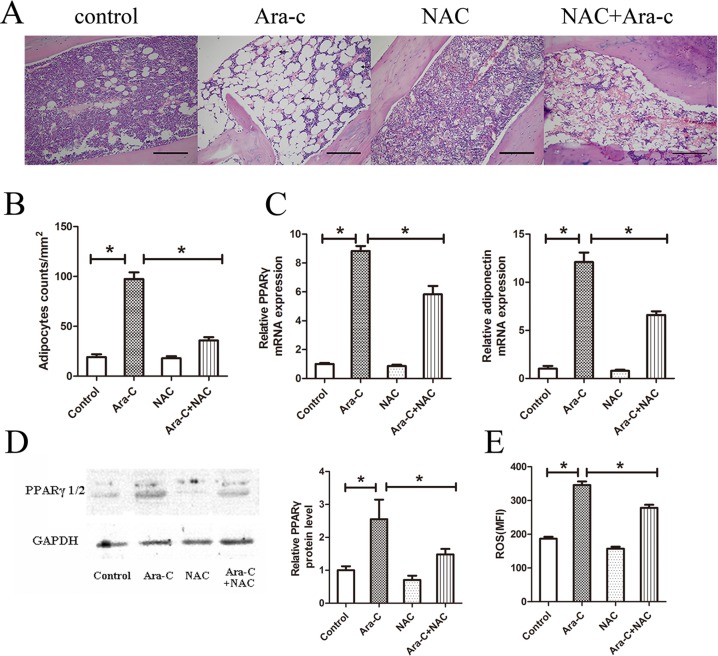
NAC diminishes adipogenesis induced by Ara-C *in vivo*. (A) BM section of the tibia from four groups on Day 7. Scale bar = 200 μm. (B) Adipocyte counts per mm^2^ in tibia BM sections. (C) Gene expression of PPARγ and adiponectin in the long bone BM on Day 7. (D) Western blot analysis of PPARγ protein on Day 7 of treatment in the long bone BM. (E) Mean fluorescence intensity of ROS in mouse BM-derived MSCs. ROS was quantified by FACS using the CM-H_2_DCFDA probe. *P < 0.05.

Our group has previously investigated adipogenesis induced by Ara-C and the effect of PPARγ inhibitor on hematopoietic recovery after chemotherapy [9]. As ROS mediate adipocyte differentiation *in vitro* and adipocytes in BM are derived from the differentiation of MSCs, whether the antioxidant NAC diminishes adipogenesis induced by Ara-C treatment was further examined in the current study. Compared to the control group, adipocyte hyperplasia and a significant increase in adipocyte counts was observed in the tibias of Ara-C-treated mice, in accordance with the results of our previous study. Furthermore, adipogenesis in tibias following Ara-C treatment was obviously inhibited by NAC (Fig. 2A-B). In addition, in the same samples, over-expression of PPARγ and adiponectin, as measured by qPCR, was suppressed by NAC treatment (Fig. 2C). Meanwhile, the protein level of PPARγ, which was increased by Ara-C treatment, was similarly decreased after NAC exposure (Fig. 2D-E). These data demonstrate that adipogenesis induced by Ara-C can be inhibited by the antioxidant NAC.

Next, the levels of ROS in mouse BM-derived MSCs were examined. Flow cytometry analysis revealed that Ara-C significantly increased the mean fluorescence intensity (MFI) of treated cells compared to the control group. In addition, this increase was attenuated by treatment with the antioxidant NAC (Fig. 2E). However, treatment with NAC alone had no effect on reducing the basal production of ROS, possibly because of the low basal level of intracellular ROS in the normal cell status. These data suggest that an increase in intracellular ROS level was induced by Ara-C and could be abolished by the antioxidant NAC.

### The potential mechanism for the elevated ROS levels

Since ROS were found to mediate adipocyte differentiation of MSCs *in vitro* and *in vivo*, the potential mechanism for the elevated ROS levels induced by Ara-C in MSCs was further investigated. Enhanced generation and/or reduced scavenging may result in increased ROS levels.

**Fig 3:**
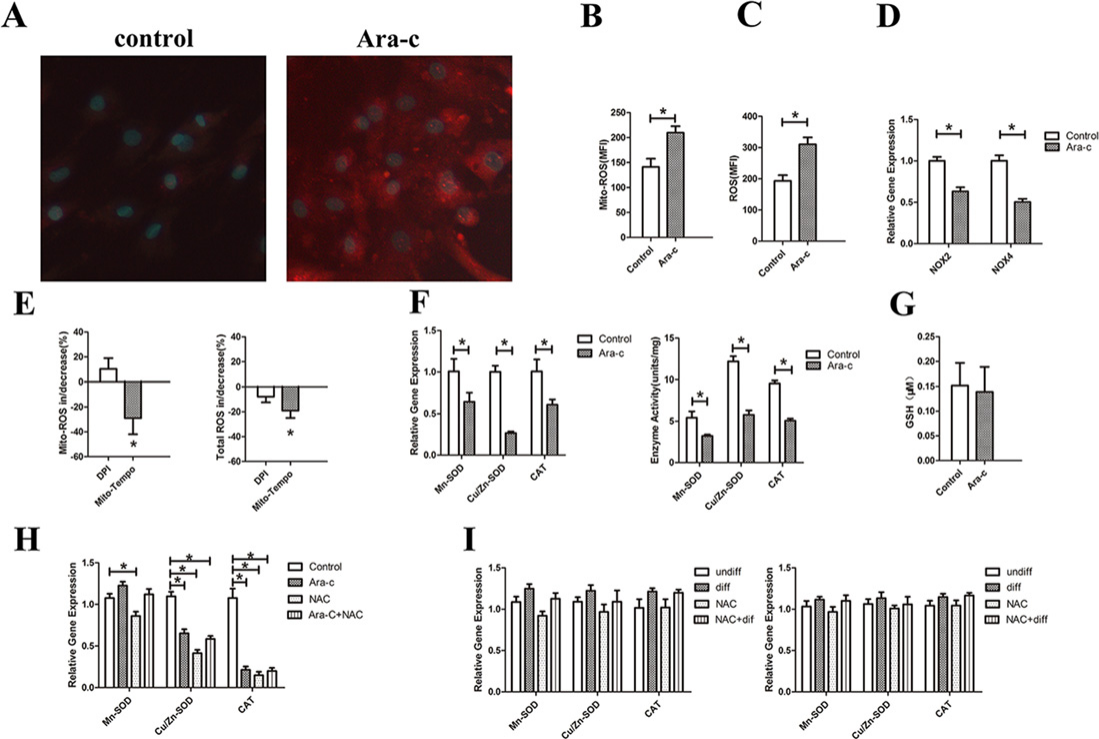
The potential mechanism for the elevated ROS levels. (A) Detection of mitochondrial ROS in live cells. Live MSCs were treated with Ara-C (100 mM for 24 hours) or without. Red fluorescence indicates the presence of mitochondrial ROS, while blue fluorescence indicates nuclei stained with Hoechest 33342. (B) Median Fluorescence Intensity (MFI) of mitochondrial ROS in the control and Ara-C groups. (C) MFI of total ROS measured by the CM-H_2_DCFDA probe in the control and Ara-C groups. (D) Gene expression of NOX2 and NOX4 in the control and Ara-C groups. (E) The mean increase/decrease of mitochondrial ROS and total ROS in Ara-C cells treated with DPI or Mito-Tempo. (F) The expression and activity of major antioxidant enzymes, including Cu/Zn-SOD, Mn-SOD and CAT. (G) The level of the antioxidant molecule GSH in the control and Ara-C groups. (H) The expression of major antioxidant enzymes *in vivo*. (I) The expression of major antioxidant enzymes on Day 7 and Day 14 of MSC differentiation induced by adipose differentiation medium. *P < 0.05.

First, ROS generation induced by Ara-C was investigated. Intracellular ROS are often generated via the catalytic action of NOX and/or the mitochondrial respiratory chain, which is cell type-dependent. Using MitoSOX, a probe that selectively detects mitochondrial superoxide, it was found that mitochondrial ROS were significantly increased in MSCs treated with Ara-C compared with the control group (Fig. 3A-B). Meanwhile, the total ROS were also significantly increased in the Ara-C group. As shown in Fig. 3C, Ara-C treated cells exhibited an approximate 60% increase in total ROS compared to the control, while mito-SOX exhibited a 48% increase in total ROS compared to the control. To examine the involvement of NOX in the rise of ROS levels induced by Ara-C, real-time PCR was performed to evaluate the NOX isoforms NOX2 and NOX4, which are the main catalytic subunits of the NOX family, in MSCs treated with (100 μM for 24 h) or without Ara-C. As shown in Fig. 3D, expression of both NOX2 and NOX4 was significantly lower in the Ara-C treated- group than in the control. Mitochondrial ROS is another source of intracellular ROS. To further dissect the respective role of NOX and mitochondria as the ROS generator, Ara-C-treated cells were also exposedto DPI and Mito-Tempo, respectively. DPI at the dose used in our experiment (500 nM) was found to block NOX enzymes without affecting mitochondrial ROS production [19], while Mito-Tempo is a well-known mitochondria-targeted antioxidant [13]. As shown in Fig. 3E, only Mito-Tempo led to a significant reduction in mitochondrial ROS and total ROS production. These data indicate that mitochondria, not NOX, are responsible for the over-generation of ROS induced by Ara-C.

Suppression of antioxidant enzymes and molecules can also lead to increased cellular ROS levels. The expression and activity of major antioxidant enzymes, including Mn-SOD, Cu/Zn-SOD and CAT, were assessed by qPCR. A significant reduction in antioxidant enzymes was observed in the Ara-C-treated group compare to control (Fig. 3F). There was no difference in the level of the antioxidant molecule GSH between the control or Ara-C groups (Fig. 3G). These data suggest that reduction of antioxidant enzymes also contributes to elevated ROS levels in MSCs treated with Ara-C.

As the antioxidant enzymes were decreased in MSCs treated with Ara-C *in vitro*, the change in antioxidant enzymes was also investigated *in vivo* using mice BM-derived MSCs exposed to Ara-C treatment. In addition, MSCs submitted to adipogenesis differentiation *in vitro* using the Adipocyte Differentiation Kit were also investigated for any change in antioxidant enzymes. Thus, the expression of antioxidant enzymes was measured by qPCR and their relative mRNA levels evaluated. As shown in Fig. 3H, compared to the control group, the expression of Cu/Zn-SOD and CAT were significantly reduced in BM-derived MSCs treated by Ara-C, NAC and Ara-C+NAC, while the expression of Mn-SOD was only significantly changed by NAC treatment. However, there was no difference in the expression of these three antioxidant enzymes in Ara-C, NAC or Ara-C+NAC treatment groups. Interestingly, these results were consistent with those obtained in MSCs *in vitro* and suggest that the reduction in antioxidant enzymes may contribute to elevate ROS levels in the Ara-C treatment group. The expression of antioxidant enzymes during MSC differentiation induced by adipose differentiation medium was also detected. There was no significant difference in the Mn-SOD, Cu/Zn-SOD or CAT gene expression among the different treatment groups at Day 7 or Day 14, which may be due to the balance of ROS generation and antioxidant levels in cells undergoing adipogenesis (Fig. 3I).

### Mito-Tempo diminishes adipocyte differentiation

**Fig 4:**
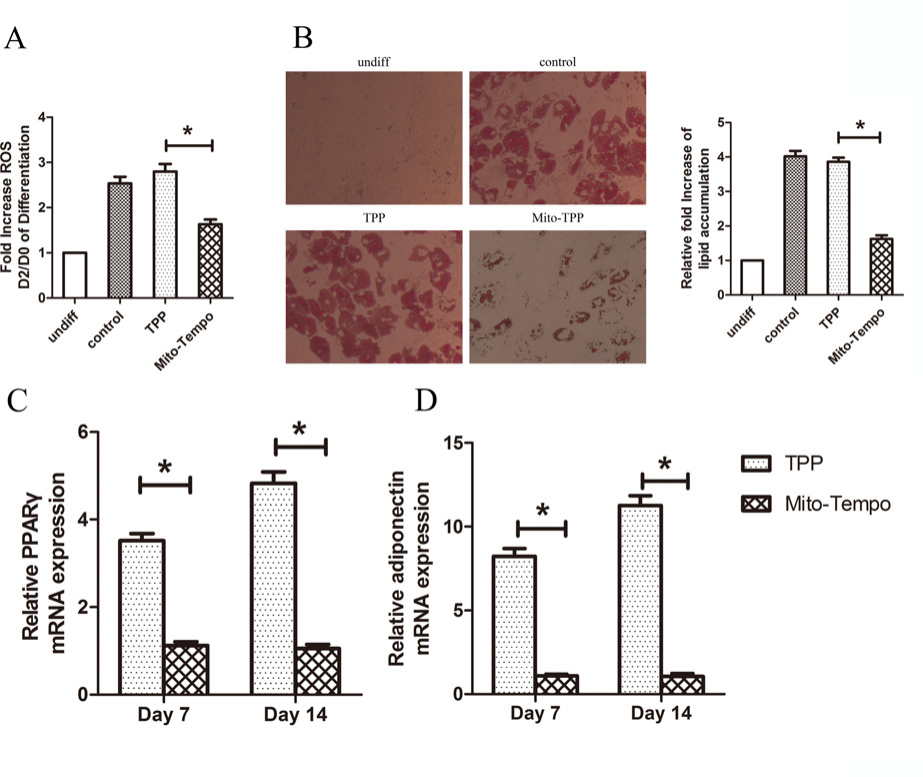
Mito-Tempo diminished adipocyte differentiation. (A) ROS levels in human MSCs on Day 0 and Day 2 of differentiation. Cells were treated with 500 nM of TPP or Mito-Tempo for 4 h prior to measurement on Day 2. (B) Mito-Tempo diminished lipid accumulation. Human MSCs were treated with 500 nM of Mito-Tempo starting on Day 2 of differentiation. Cells were fixed and stained with oil red O on Day 14. The oil red-O was extracted with isopropanol and absorbance was measured at 518 nm. (C, D) Gene expression of PPARγ and adiponectin was decreased in the presence of Mito-Tempo on Day 7 and Day 14 of differentiation.*P < 0.05.

To further specify the role of ROS produced by mitochondria in adipocyte differentiation, the effect of Mito-Tempo on adipocyte differentiation of MSCs was investigated *in vitro*. The Mito-Tempo antioxidant is a combination of piperidine nitroxide and the lipophilic cation triphenylphosphonium (TPP), giving Mito-Tempo the ability to pass through lipid bilayers with ease and accumulate several hundred-fold inside the mitochondria. Human MSCs were treated with control TPP and Mito-Tempo starting on Day 2 of differentiation. As shown in Fig. 4A, the increase in ROS in the early stage of differentiation was attenuated in the presence of Mito-Tempo. Furthermore, Mito-Tempo significantly reduced lipid accumulation (Fig. 4B) as well as the expression of PPARγ and adiponectin (Fig. 4C-D). These results demonstrate that mitochondrial ROS are required for adipocyte differentiation of MSCs.

## Discussion

As an element of the hematopoietic niche, the adipocyte has historically been considered as merely a passive storage tissue filling marrow cavities [20]. More recently, several studies revealed that adipocytes in the niche may play a negative role in hematopoiesis, while antagonizing adipogenesis in the BM may promote hematopoietic recovery. In lipoatrophic A-ZIP/F1 “fatless” mice, which are genetically incapable of generating adipocytes, treatment with the PPARγ inhibitor BADGE inhibits adipogenesis induced by irradiation or chemotherapy, thus accelerating marrow engraftment compared to wild type or untreated mice [7]. In addition, as shown in recent real-time imaging studies *in vitro* [21] and *in vivo* [22], hematopoiesis is maintained in balance by support from osteoblasts and suppression by adipocytes. If BM undergoes stress (such as irradiation or chemotherapy) or senesces, adipocytes predominantly suppress hematopoiesis. Therefore, the increase in adipocytes induced by chemotherapeutic drugs may play a negative role in hematopoietic recovery following chemotherapy and HSCT. As adipocytes in BM are derived from the differentiation of MSCs, the current study was focused on the effect of the chemotherapy drug Ara-C on adipocyte differentiation.

It has been shown that ROS take part in a variety of processes, including cell motility, differentiation, cell-cycle progression and growth factor signaling [11]. Chronic elevated levels of ROS within a cell have been attributed to disease, including chronic lymphocyte leukemia [23], acute myeloid leukemia [24], obesity [25] and diabetes [26]. Interestingly, several studies have demonstrated that ROS levels are increased during adipogenesis [14,27,28]. Moreover, recent studies have revealed that the increase in intracellular ROS not only acts as byproducts of the differentiation process, but also may be involved in mediating differentiation of MSCs into adipocytes [12,13]. Therefore, the role of ROS in adipogenesis *in vitro* and *in vivo* was investigated. In our current study, an increase in intracellular ROS was observed during the early stage of adipocyte differentiation, accompanied by over-expression of the transcription factor PPARγ, which is required to initiate adipocyte differentiation. The use of the antioxidant NAC effectively inhibited the increase in ROS and the expression of PPARγ. By using a mouse adipocyte hyperplasia model, the interactions between ROS generation and chemotherapy-induced adipogenesis were examined. Our results demonstrate that Ara-C-induced adipogenesis is associated with an increase in intracellular ROS level, as the antioxidant NAC could diminish adipogenesis via scavenging of ROS induced by Ara-C exposure. These results indicate that ROS are involved in adipogenesis induced by hematopoiesis stress. However, the blocking effects of NAC on ROS and adipogenesis are not significant in homeostatic conditions, which may due to the low basal level of ROS in homeostasis. Hence, integrating *in vivo* and *in vitro* results, the elevation of ROS induced by hematopoiesis stress may participate in the activation of PPARγ transcriptional machinery during adipocyte differentiation of MSCs, thereby leading to adipogenesis in the BM.

Furthermore, there is an inverse relationship between adipogenic differentiation and osteogenic differentiation [29], while the mechanisms controlling the differentiation of MSCs are not fully clear. Interestingly, different levels of ROS may regulate osteoblast differentiation versus adipocyte differentiation of MSCs[13,30]. The fate of MSCs, especially for adipogenic differentiation, seems to be regulated by PPARγ, a type of nuclear hormone receptor [31]. The activation of PPARγ promotes adipogenesis as well as inhibits osteogenesis [32]. Our results clearly suggest that the increase in ROS may contribute to the PPARγ transcriptional machinery and then promote adipogenesis after Ara-C treatment. In accordance with previous reports, intracellular ROS levels drive MSCs toward adipocyte differentiation by regulating specific transcription factors [12,13]. On the other hand, it has been reported that a dramatic decrease in intracellular ROS was observed during osteogenic differentiation of human MSCs [30]. Moreover, osteoblast differentiation at the expense of adipocyte differentiation was shown to be mediated by hypoxia-inducible factor 1-alpha in human stromal precursors [33]. Taken together, we suggest that ROS may play a role in regulating the differentiation direction of MSCs.

Because the mechanism responsible for the elevation of ROS levels in MSCs treated with Ara-C and the differentiation process of MSCs remains unclear, the mechanism was investigated in our experimental models. Enhanced generation and/or reduced scavenging may result in an increase in cellular ROS generation. Our results demonstrate that mitochondria, but not the NOX family, is the main source of ROS production in MSCs treated with Ara-C. Furthermore, the use of the mitochondrial targeted antioxidant Mito-Tempo significantly diminished adipocyte differentiation. However, the source of ROS may be specific due to different cell types and conditions. Knocking down NOX4 by RNA interference inhibited ROS production and adipocyte differentiation in a murine MSC line, which was treated with differentiation-inducing agents [12]. Another study has reported that although NOX4 generated ROS leading to the regulation of proliferation and migration of adipose-derived MSCs, the use of NOX4 inhibitor or NOX4 silencing alone was not sufficient to inhibit hypoxia-induced adipocyte differentiation[34]. However, the modulation of mitochondrial ROS increased in hypoxia as mitochondrial ROS scavengers significantly reduced hypoxia-induced adipocyte differentiation of adipose-derived MSCs [34]. Indeed, Carriere et al. found that pharmacologic inhibition of complex I and III of the mitochondrial respiratory chain by inhibitors, such as rotenone and antimycin-A, reduced adipocyte differentiation of 3T3-F442A pre-adipocytes and adipose-derived stromal cells by triggering hypoxia-dependent inhibition of adipose differentiation [35,36]. The group described a specific regulation by mitochondrial ROS of the expression of the adipogenic repressor CHOP-10/GADD153, considered as anti-adipogenic signaling molecules [35,36]. Importantly, Tormos et al. found that antimycin-A, which is known to diminish respiratory chain function and to maintain superoxide production from complex III [37], did not increase the PPARγ-dependent gene targets at Day 7 of human MSCs differentiation into adipocytes [13]. Accordingly with our results, mitochondrial-targeted antioxidants abolished the increase in PPARγ-mediated transcription in the presence of antimycin A [13]. All of these results clearly demonstrate that mitochondrial ROS are a positive regulator of adipocyte differentiation. However, the specific and in-depth mechanism of how ROS regulate MSC differentiation still needs to be investigated further. Interestingly, the regulation of cellular redox state involved in this MSC differentiation, did not only concerns ROS generation but also the cellular level of antioxidant systems.

The reduction in the main cellular antioxidant enzymes, Mn-SOD, Cu/Zn-SOD and CAT, may also contribute to the increase in ROS in MSCs treated with Ara-C, even if this reduction was not observed in MSCs treated with the Adipogenesis Differentiation Kit. Interestingly, the changes in antioxidant enzymes and molecules during adipocyte differentiation of 3T3-L1 preadipocytes, human BM-derived MSCs, and human adipose-derived stem cells seem complex due to the various results obtained in different studies [30,38–40]. However, we speculate that there is a balance between ROS generation and antioxidant enzymes in cells undergoing adipogenesis. The different treatments used to induce this differentiation, the various detection methods used to measure adipogenesis, ROS generation methods, cell antioxidant systems and the diversity of cell types used in these investigations may contribute to complicate the understanding of the cellular signaling pathways involved.

In summary, the present study demonstrates that ROS are required to participate in the activation of PPARγ transcriptional machinery during adipogenic differentiation of MSCs, which is induced by Ara-C, while NAC inhibits adipogenesis through decreasing intracellular ROS levels. Mitochondrial ROS are required for adipocyte differentiation and responsible for the over-generation of ROS induced by Ara-C. Reduction in cellular antioxidant enzymes may also contribute to elevate levels of ROS in MSCs treated with Ara-C. Our data suggest that inhibition of ROS may improve hematopoietic recovery after long-term chemotherapy and HSCT, which may be a potential therapeutic target in the clinic.

## References

[1] Lee KH, Lee JH, Choi SJ, Kim S, Seol M, Lee YS, et al Failure of trilineage blood cell reconstitution after initial neutrophil engraftment in patients undergoing allogeneic hematopoietic cell transplantation—frequency and outcomes. Bone Marrow Transplant. 2004 33: 729–734. 14755315 10.1038/sj.bmt.1704428

[2] Damon LE, Rugo HS, Ries CA, Linker CA Post-remission cytopenias following intense induction chemotherapy for acute myeloid leukemia. Leukemia. 1994 8: 535–541. 8152248

[3] Kong Y, Chang YJ, Wang YZ, Chen YH, Han W, Wang Y, et al Association of an impaired bone marrow microenvironment with secondary poor graft function after allogeneic hematopoietic stem cell transplantation. Biol Blood Marrow Transplant. 2013 19: 1465–1473. 10.1016/j.bbmt.2013.07.014 23879970

[4] del Canizo MC, Lopez N, Vazquez L, Fernandez ME, Ana B, Caballero MD, et al Hematopoietic damage prior to PBSCT and its influence on hematopoietic recovery. Haematologica. 1999 84: 511–516. 10366794

[5] Domenech J, Gihana E, Dayan A, Truglio D, Linassier C, Desbois I, et al Haemopoiesis of transplanted patients with autologous marrows assessed by long-term marrow culture. Br J Haematol. 1994 88: 488–496. 7819062 10.1111/j.1365-2141.1994.tb05064.x

[6] del Canizo C, Lopez N, Caballero D, Fernandez E, Brufau A, Vazquez L, et al Haematopoietic damage persists 1 year after autologous peripheral blood stem cell transplantation. Bone Marrow Transplant. 1999 23: 901–905. 10338045 10.1038/sj.bmt.1701730

[7] Naveiras O, Nardi V, Wenzel PL, Hauschka PV, Fahey F, Daley GQ Bone-marrow adipocytes as negative regulators of the haematopoietic microenvironment. Nature. 2009 460: 259–263. 10.1038/nature08099 19516257 PMC2831539

[8] Belaid-Choucair Z, Lepelletier Y, Poncin G, Thiry A, Humblet C, Maachi M, et al Human bone marrow adipocytes block granulopoiesis through neuropilin-1-induced granulocyte colony-stimulating factor inhibition. Stem Cells. 2008 26: 1556–1564. 10.1634/stemcells.2008-0068 18388301

[9] Zhu RJ, Wu MQ, Li ZJ, Zhang Y, Liu KY Hematopoietic recovery following chemotherapy is improved by BADGE-induced inhibition of adipogenesis. Int J Hematol. 2013 97: 58–72. 10.1007/s12185-012-1233-4 23264188

[10] Rosen ED, Spiegelman BM PPARgamma: a nuclear regulator of metabolism, differentiation, and cell growth. J Biol Chem. 2001 276: 37731–37734. 11459852 10.1074/jbc.R100034200

[11] Valko M, Leibfritz D, Moncol J, Cronin MT, Mazur M, Telser J Free radicals and antioxidants in normal physiological functions and human disease. Int J Biochem Cell Biol. 2007 39: 44–84. 16978905 10.1016/j.biocel.2006.07.001

[12] Kanda Y, Hinata T, Kang SW, Watanabe Y Reactive oxygen species mediate adipocyte differentiation in mesenchymal stem cells. Life Sci. 2011 89: 250–258. 10.1016/j.lfs.2011.06.007 21722651

[13] Tormos KV, Anso E, Hamanaka RB, Eisenbart J, Joseph J, Kalyanaraman B, et al Mitochondrial complex III ROS regulate adipocyte differentiation. Cell Metab. 2011 14: 537–544. 10.1016/j.cmet.2011.08.007 21982713 PMC3190168

[14] Lee H, Lee YJ, Choi H, Ko EH, Kim JW Reactive oxygen species facilitate adipocyte differentiation by accelerating mitotic clonal expansion. J Biol Chem. 2009 284: 10601–10609. 10.1074/jbc.M808742200 19237544 PMC2667747

[15] Carew JS, Zhou Y, Albitar M, Carew JD, Keating MJ, Huang P Mitochondrial DNA mutations in primary leukemia cells after chemotherapy: clinical significance and therapeutic implications. Leukemia. 2003 17: 1437–1447. 12886229 10.1038/sj.leu.2403043

[16] Hu L, Cheng H, Gao Y, Shi M, Liu Y, Hu Z, et al Antioxidant N-acetyl-l-cysteine increases engraftment of human hematopoietic stem cells in immune-deficient mice. Blood. 2014 124: e45–48. 10.1182/blood-2014-03-559369 25287706 PMC4231425

[17] Azizi SA, Stokes D, Augelli BJ, DiGirolamo C, Prockop DJ Engraftment and migration of human bone marrow stromal cells implanted in the brains of albino rats—similarities to astrocyte grafts. Proc Natl Acad Sci U S A. 1998 95: 3908–3913. 9520466 10.1073/pnas.95.7.3908 PMC19936

[18] Gagnon A, Lau S, Sorisky A Rapamycin-sensitive phase of 3T3-L1 preadipocyte differentiation after clonal expansion. J Cell Physiol. 2001 189: 14–22. 11573200 10.1002/jcp.1132

[19] Regina Jitschin ADH, 1 Heiko Bruns,1 Andreas Gießl,2 Juliane Bricks,1 Jana Berger,1 Domenica Saul,1, Michael J. Eckart AM, 1 and Dimitrios Mougiakakos1 Mitochondrial metabolism contributes to oxidative stress and reveals therapeutic targets in chronic lymphocytic leukemia. Blood. 2014. 10.1182/blood-2013-10-532200 24553174

[20] Gimble JM, Robinson CE, Wu X, Kelly KA The function of adipocytes in the bone marrow stroma: an update. Bone. 1996 19: 421–428. 8922639 10.1016/s8756-3282(96)00258-x

[21] Xie Y, Yin T, Wiegraebe W, He XC, Miller D, Stark D, et al Detection of functional haematopoietic stem cell niche using real-time imaging. Nature. 2008 457: 97–101. 10.1038/nature07639 19052548

[22] Lo Celso C, Fleming HE, Wu JW, Zhao CX, Miake-Lye S, Fujisaki J, et al Live-animal tracking of individual haematopoietic stem/progenitor cells in their niche. Nature. 2009 457: 92–96. 10.1038/nature07434 19052546 PMC2820276

[23] Jitschin R, Hofmann AD, Bruns H, Giessl A, Bricks J, Berger J, et al Mitochondrial metabolism contributes to oxidative stress and reveals therapeutic targets in chronic lymphocytic leukemia. Blood. 2014 123: 2663–2672. 10.1182/blood-2013-10-532200 24553174

[24] Hole PS, Darley RL, Tonks A Do reactive oxygen species play a role in myeloid leukemias? Blood. 2011 117: 5816–5826. 10.1182/blood-2011-01-326025 21398578

[25] Furukawa S, Fujita T, Shimabukuro M, Iwaki M, Yamada Y, Nakajima Y, et al Increased oxidative stress in obesity and its impact on metabolic syndrome. J Clin Invest. 2004 114: 1752–1761. 15599400 10.1172/JCI21625 PMC535065

[26] Houstis N, Rosen ED, Lander ES Reactive oxygen species have a causal role in multiple forms of insulin resistance. Nature. 2006 440: 944–948. 16612386 10.1038/nature04634

[27] Imhoff BR, Hansen JM Extracellular redox environments regulate adipocyte differentiation. Differentiation. 2010 80: 31–39. 10.1016/j.diff.2010.04.005 20471742

[28] Wilson-Fritch L, Burkart A, Bell G, Mendelson K, Leszyk J, Nicoloro S, et al Mitochondrial biogenesis and remodeling during adipogenesis and in response to the insulin sensitizer rosiglitazone. Mol Cell Biol. 2003 23: 1085–1094. 12529412 10.1128/MCB.23.3.1085-1094.2003 PMC140688

[29] Bethel M, Chitteti BR, Srour EF, Kacena MA The changing balance between osteoblastogenesis and adipogenesis in aging and its impact on hematopoiesis. Curr Osteoporos Rep. 2013 11: 99–106. 10.1007/s11914-013-0135-6 23423562 PMC3643998

[30] Chen CT, Shih YR, Kuo TK, Lee OK, Wei YH Coordinated changes of mitochondrial biogenesis and antioxidant enzymes during osteogenic differentiation of human mesenchymal stem cells. Stem Cells. 2008 26: 960–968. 10.1634/stemcells.2007-0509 18218821

[31] Rosen ED, Sarraf P, Troy AE, Bradwin G, Moore K, Milstone DS, et al PPAR gamma is required for the differentiation of adipose tissue in vivo and in vitro. Mol Cell. 1999 4: 611–617. 10549292 10.1016/s1097-2765(00)80211-7

[32] Nuttall M Controlling the balance between osteoblastogenesis and adipogenesis and the consequent therapeutic implications. Current Opinion in Pharmacology. 2004 4: 290–294. 15140422 10.1016/j.coph.2004.03.002

[33] Wagegg M, Gaber T, Lohanatha FL, Hahne M, Strehl C, Fangradt M, et al Hypoxia promotes osteogenesis but suppresses adipogenesis of human mesenchymal stromal cells in a hypoxia-inducible factor-1 dependent manner. PLoS One. 2012 7: e46483 10.1371/journal.pone.0046483 23029528 PMC3459928

[34] Kim JH, Kim SH, Song SY, Kim WS, Song SU, Yi T, et al Hypoxia induces adipocyte differentiation of adipose-derived stem cells by triggering reactive oxygen species generation. Cell Biol Int. 2014 38: 32–40. 10.1002/cbin.10170 23956071

[35] Carriere A, Ebrahimian TG, Dehez S, Auge N, Joffre C, Andre M, et al Preconditioning by mitochondrial reactive oxygen species improves the proangiogenic potential of adipose-derived cells-based therapy. Arterioscler Thromb Vasc Biol. 2009 29: 1093–1099. 10.1161/ATVBAHA.109.188318 19423864

[36] Carriere A, Carmona MC, Fernandez Y, Rigoulet M, Wenger RH, Penicaud L, et al Mitochondrial reactive oxygen species control the transcription factor CHOP-10/GADD153 and adipocyte differentiation: a mechanism for hypoxia-dependent effect. J Biol Chem. 2004 279: 40462–40469. 15265861 10.1074/jbc.M407258200

[37] Turrens JF, Alexandre A, Lehninger AL Ubisemiquinone is the electron donor for superoxide formation by complex III of heart mitochondria. Arch Biochem Biophys. 1985 237: 408–414. 2983613 10.1016/0003-9861(85)90293-0

[38] Calzadilla P, Sapochnik D, Cosentino S, Diz V, Dicelio L, Calvo JC, et al N-acetylcysteine reduces markers of differentiation in 3T3-L1 adipocytes. Int J Mol Sci. 2011 12: 6936–6951. 10.3390/ijms12106936 22072928 PMC3211019

[39] Lee OH, Seo MJ, Choi HS, Lee BY Pycnogenol(R) inhibits lipid accumulation in 3T3-L1 adipocytes with the modulation of reactive oxygen species (ROS) production associated with antioxidant enzyme responses. Phytother Res. 2012 26: 403–411. 10.1002/ptr.3568 21796705

[40] Higuchi M, Dusting GJ, Peshavariya H, Jiang F, Hsiao ST, Chan EC, et al Differentiation of human adipose-derived stem cells into fat involves reactive oxygen species and Forkhead box O1 mediated upregulation of antioxidant enzymes. Stem Cells Dev. 2013 22: 878–888. 10.1089/scd.2012.0306 23025577 PMC3585477

